# Patients accept therapy using embryonic stem cells for Parkinson’s disease: a discrete choice experiment

**DOI:** 10.1186/s12910-023-00966-1

**Published:** 2023-10-12

**Authors:** Karin Schölin Bywall, Jennifer Drevin, Catharina Groothuis-Oudshoorn, Jorien Veldwijk, Dag Nyholm, Hakan Widner, Trinette van Vliet, Elena Jiltsova, Mats Hansson, Jennifer Viberg Johansson

**Affiliations:** 1 School of Health, Care and Social Welfare, Division of Health and Welfare Technology, Mälardalen University, Västerås, Sweden; 2 Centre for Research Ethics and Bioethics, Uppsala University, Box 564, Uppsala, SE-751 22 Sweden; 3 Health Technology and Services Research (HTSR), Faculty of Behavioural Management and Social Sciences, University of Twente, Enschede, The Netherlands; 4 Erasmus School of Health Policy & Management, Erasmus University Rotterdam, Rotterdam, The Netherlands; 5 Erasmus Choice Modelling Centre, Erasmus University Rotterdam, Rotterdam, The Netherlands; 6 Department of Medical Sciences, Uppsala University, Uppsala University Hospital, Uppsala, SE-751 85 Sweden; 7 Department of Neurology, Skåne University Hospital, Lund, SE-221 85 Sweden; 8 The Institute for Future Studies, Holländargatan 13, 111 36, Stockholm, Sweden

**Keywords:** Ethics, Human embryonic stem cells, Parkinson’s disease, Patient preferences

## Abstract

**Background:**

New disease-modifying ways to treat Parkinson’s disease (PD) may soon become a reality with intracerebral transplantation of cell products produced from human embryonic stem cells (hESCs). The aim of this study was to assess what factors influence preferences of patients with PD regarding stem-cell based therapies to treat PD in the future.

**Methods:**

Patients with PD were invited to complete a web-based discrete choice experiment to assess the importance of the following attributes: (i) type of treatment, (ii) aim of treatment, (iii) available knowledge of the different types of treatments, (iv) effect on symptoms, and (v) risk for severe side effects. Latent class conditional logistic regression models were used to determine preference estimates and heterogeneity in respondents’ preferences.

**Results:**

A substantial difference in respondents’ preferences was observed in three latent preference patterns (classes). “Effect on symptoms” was the most important attribute in class 1, closely followed by “type of treatment,” with medications as preferred to other treatment alternatives. Effect on symptoms was also the most important attribute in class 2, with treatment with hESCs preferred over other treatment alternatives. Likewise for class 3, that mainly focused on “type of treatment” in the decision-making. Respondents’ class membership was influenced by their experience in treatment, side effects, and advanced treatment therapy as well as religious beliefs.

**Conclusions:**

Most of the respondents would accept a treatment with products emanating from hESCs, regardless of views on the moral status of embryos. Preferences of patients with PD may provide guidance in clinical decision-making regarding treatments deriving from stem cells.

**Supplementary Information:**

The online version contains supplementary material available at 10.1186/s12910-023-00966-1.

## Background

Parkinson’s disease (PD) is the most common serious movement disorder in the world, affecting about 1% of adults older than 60 years [1]. Being diagnosed with PD will change a person’s life, as the disease is characterized by progressive development of complex motor and non-motor symptoms [2]. Symptomatic progression is inevitable, yet unpredictable [3]. Currently, medicines and allied treatments offer only symptomatic relief. Such treatments aim at increasing patients’ quality of life and functional capacity [4]. Deep brain stimulation surgery may be an alternative when medication and physiotherapy do not give a sustained effect or if unbearable side effects appear [5]. Today there is no treatment available that can modify or stop the progression of the disease [6].

New and disease-modifying ways to treat PD and repair damage caused by PD may soon become a reality [7]. Embryonic stem cells (ESCs) have the ability to self-renew and reprogram, allowing the derivation of any adult differentiated cell type [8]. Cell transplantation of human embryonic stem cells (hESCs), derived from surplus embryos donated by couples who have undergone in vitro fertility (IVF) treatment, have been transplanted to experimental animals with models of PD resulting in symptomatic improvement and reformation of neuronal circuitries [9]. This may become a potential stem cell-based therapy with a possibility to alleviate debilitating neurodegenerative disorders like PD. Researchers are also exploring the potential of autologous transplantation of human induced pluripotent stem cells (iPS cells), derived from somatic cells [10].

Previous qualitative research has found that the general public, health care professionals, and couples who underwent fertility treatments were positive to research on leftover embryos to derive treatment with hESCs [11–13]. Nevertheless, the use of human embryos in cell-based therapy is associated with several ethical and legal issues. Patients who believe that human embryos are subjects with rights may be against the destruction of embryos to derive treatment with hESCs, whereas patients viewing the embryo as too undeveloped to have such a moral status generally are expected to permit such treatment. Therefore, it is essential to quantitatively assess factors involved in preferences of patients with PD for potential stem cell-based therapies to treat PD [14]. The aim of this study was to assess what factors influence preferences of patients with PD regarding stem cell-based therapies to treat PD in the future.

## Methods

### Discrete choice experiment

Preferences of patients with PD for potential cell-based therapies to treat PD were assessed by a Discrete Choice Experiment (DCE) in Swedish patients with PD. The DCE is a cross-sectional survey method to investigate individuals preferences and can be used to determine the relative importance of different characteristics of an intervention and predict uptake of different interventions [15]. Respondents of a DCE are faced with a set of hypothetical choice questions with two or more alternatives, characterized by different characteristics (i.e., attributes) with varying levels. The DCE method also allows for the calculation of attribute trade-offs [16].

### Selection of attributes and levels

We performed a scoping literature review to identify attributes of treatments for PD that potentially were of importance for patients with PD when choosing treatment. Qualitative and quantitative papers investigating preferences of patients with PD related to treatment for PD were included. All literature searches were performed in PubMed and the keywords used were Parkinson disease, patient preferences, preferences, treatment, medication, and attributes. We identified 193 papers, including 29 papers that were relevant for this project, of which 20 papers remained after excluding duplicates. After reading the full text papers, 209 potential attributes were identified. Out of the 209 attributes identified in the scoping literature review, 115 attributes were unique. These attributes were condensed down to 45 by merging similar concepts. The identified attributes were discussed in a group consisting of a representative patient of a Parkinson patient organization, neurologists, a research coordinator, a nurse working with patients with PD, and researchers knowledgeable in DCE methodology. Based on the discussions in this group, 11 attributes remained. We let 17 patients with PD rank the 11 attributes from most to least important, for their decision about PD treatment. Based on the mean ranks of the attributes and discussions with clinicians, eight attributes remained. These were re-categorized into the five attributes that were assigned relevant levels to be assessed by the DCE: (i) type of treatment, (ii) aim of treatment, (iii) available knowledge of the different types of treatments, (iv) effect on symptoms, and (v) risk for severe side effects (Table 1).

**Table 1:** Attributes and levels included in the DCE

Attribute	Formulation	Levels	Description of levels
Type of treatment	The treatment consists of…	hESCs^a^	Cells taken from donated fertilized eggs. The cells have been multiplied and directed to produce dopamine.
		iPS cells^b^	Your own/donated cells (e.g. blood cells) that have been multiplied and directed to produce dopamine.
		Electric stimulation	An implanted electrode with thin wire and stimulator that stimulates the brain.
		Drug	Drugs for Parkinson’s disease.
Aim of treatment	The aim of the treatment is to…	Relieve symptoms	Improve function and well-being without affecting the development of the disease. As the disease progresses, doses usually need to be increased to obtain sufficient relief.
		Slow down disease progression	Affects the development of the disease so that the disease develops more slowly than it would have if you had not been treated.
		Repair damage caused by disease	Affects disease progression and restores functions lost due to your Parkinson’s disease.
Available knowledge and experience of treatment	Number of patients that have received the treatment is…	50	After clinical research studies, the treatment has been approved for treatment against Parkinson’s disease. A total of 50 people have received the treatment.
		500	After clinical research studies, the treatment has been approved for treatment against Parkinson’s disease. A total of 500 people have received the treatment.
		5000	After clinical research studies, the treatment has been approved for treatment against Parkinson’s disease. A total of 5000 people have received the treatment.
Effect on symptoms	Treatment effect on symptoms (for example, balance difficulties, tremors, depression, and dementia). The proportion that achieves sufficient function and well-being to at the moment not needing additional/different treatment for Parkinson’s is…	2 out of 10 will get enough effectiveness	Out of 10 who receive the treatment, 2 people achieve sufficient function and well-being to not currently need additional/different treatment for Parkinson’s.
		5 out of 10 will get enough effectiveness	Out of 10 who receive the treatment, 5 people achieve sufficient function and well-being to not currently need additional/different treatment for Parkinson’s.
		8 out of 10 will get enough effectiveness	Out of 10 who receive the treatment, 8 people achieve sufficient function and well-being to not currently need additional/different treatment for Parkinson’s.
Risk for severe side effects	The risk that the treatment causes a serious side effect that has a negative lasting effect on function and well-being is…	20 out of 1000	Out of 50 people who start the treatment, 1 person suffers some kind of serious side effect.
		10 out of 1000	Out of 100 people who start the treatment, 1 person suffers some form of serious side effect.
		1 out of 1000	Out of a thousand people who start the treatment, 1 person suffers some kind of serious side effect.

^a^ Human embryonic stem cells

^b^ Induced pluripotent stem cells

### Sample size and study population

We followed methodological guidelines to estimate the sample size needed to identify preferences of patients with PD and differences within those preferences [17]. We considered the number of attributes in the DCE (Table 1) and the number of choice questions for each respondent (n = 9). Based on the sample size requirements for a DCE and accounting for subgroup analysis, we aimed for a sample size of 500 respondents.

Patients with PD were recruited from neurology clinics at two university hospitals in Sweden. This study was approved by the Swedish Ethical Review Authority (Dnr 2019–06539). Information about the study was sent out by mail to all potential respondents fulfilling the inclusion criteria: patients diagnosed with PD, 18 years or older, able to read and understand Swedish. Patients with a known dementia diagnosis were excluded. Information about the study was sent out to 1266 patients. Patients who had not responded within two weeks were sent a reminder by mail. All respondents provided their informed consent before entering the survey. Two patients formally declined participation, and five patients were unable to participate due to technical or health-related restrictions. In total, 498 patients participated in the study (i.e., 39% response rate).

### Survey administration

**Fig. 1:**
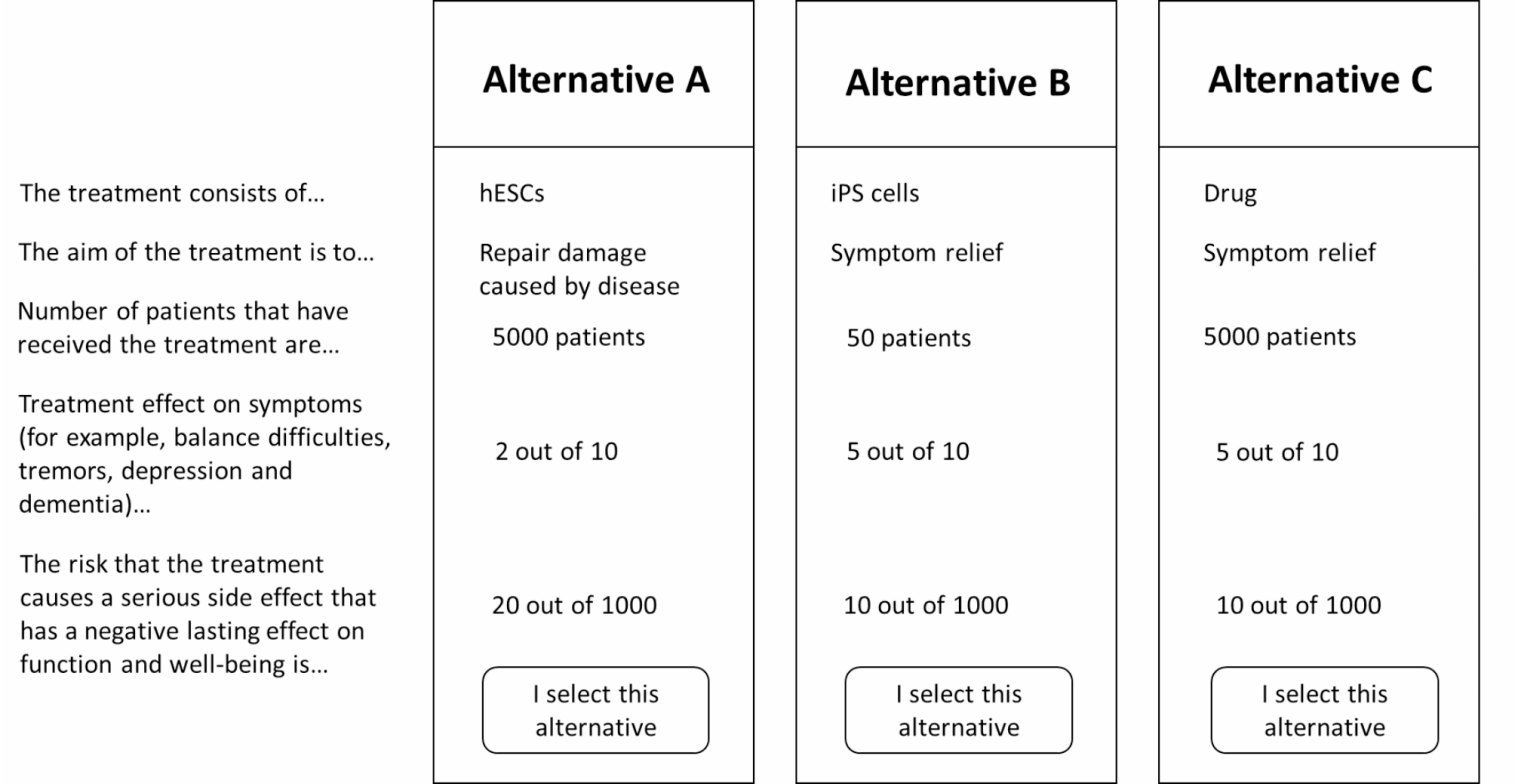
Example of a choice scenario

This survey was administered as a web-based survey that included three parts: (i) information about the attributes and levels, (ii) the DCE with hypothetical choice scenarios, and (iii) demographic and attitude questions (see supplementary file for survey). The survey was created for this study and administered using Sawtooth Software (Sawtooth Software Inc.). Each respondent was faced with nine hypothetical choice scenarios that each included three alternatives. The respondents were asked to select the alternative that they most preferred out of the three presented to them. The first two alternatives were experimentally designed to assess preferences for potential treatment alternatives for PD and the third was a fixed profile (i.e., nonexperimental) to represented standard care (drugs) for patients with PD (Fig. 1). We used a Bayesian D-efficient design to construct the choice scenarios for the DCE using the NGene program (version1.2.1; ChoiceMetrics 2012). Prior information on the attribute importance was gathered from a pilot test (n = 142) in patients with PD. The design used 500 Halton draws and 1000 repetitions. Using the pilot data, a multinomial logit (MNL) model was fitted, and the beta estimates was used as priors for the final experimental DCE design.

Some conditions were posted on the design: if the aim of treatment was to repair damage caused by disease, the treatment could not consist of ‘electric stimulation’ or ‘drug’. If the aim of the treatment was to slow down disease progression, the treatment could not consist of ‘electric stimulation’. The final discrete choice survey consisted of 36 unique choice scenarios divided into four blocks; each respondent was randomized into one block and answered nine choice scenarios. The choice questions also included a hover function with further explanations of the attributes and the levels (see Table 1 for full description of the attribute levels).

The demographical and attitude questions included background questions (e.g., age, gender, and education) and disease-related questions (e.g., disease duration, treatment, and side effects). Moreover, the respondents’ attitudes were gauged with a ranking exercise with eight statements that they were asked to place in the order they found most important. The attitude questions asked respondents about their moral stands on the status of an embryo, and a ranking exercise to prioritize eight statements.

The respondents were asked about their views on how to regard the products left over after IVF procedures, which may be used for hESC isolation, that is, the blastocyst. Whether this material was regarded as a lump of cells or “something more” was used to dichotomize the answers. Questions to assess respondents’ health literacy [18] and health numeracy [19] were also included to define the sample.

### Statistical analyses

The statistical analyses, in particular the estimation of the latent class model were performed using R 4.0.2 (R Core Team, 2018), the mlogit (version 1.1-1; Yves Croissant, 2009) and the gmnl (version 1.1–3.3; Mauricio Sarrias, 2017) [20].

Demographics describing the population’s age, gender, country of birth, occupational situation, education, health numeracy, health literacy, drug frequency, disease duration, number of experienced side effects, and experience of advanced treatment were presented in mean, median, and percentages. The overall level of health literacy and numeracy was calculated for each respondent. Individuals who responded “strongly disagree” or “disagree” to one of the items were categorized as having low health literacy. Individuals who responded with “neither agree nor disagree” with one of the items were categorized as having medium health literacy. Individuals responding “agree” or “strongly agree” to all the items were categorized as having high health literacy, and likewise for numeracy.

Respondents’ attitude toward the moral status of a couple of days’ old human embryo was assessed using this question: “The human is perceived to have a special moral position, in the sense of having rights just by being human. What moral position does a human embryo that is only a few days old have?” The respondents had four statements from which to select: (1) “The embryo is just a lump of cells; it is meaningless to discuss its moral status,” (2) “The embryo has a moral status that is in between being just a lump of cells and being a human being,” (3) “The embryo in its moral status is closer to being a human than just a lump of cells,” and (4) “The embryo has the same moral status as a human being.” The variable was dichotomized based on the frequency of the data. Respondents answering “The embryo is just a lump of cells; it is meaningless to discuss its moral status” formed one group, and the rest another group. One-way analysis of variance and nonparametric measures were used to test the differences between the personal characteristics and the different perceptions of whether an embryo is more than a lump or cells or not.

The most important attitudinal statement was given a 1, the second most important the number 2 and so forth. The ranking exercise was illustrated with a boxplot by the median value of each statement, stratified on the different perceptions of whether an embryo is more than a lump of cells.

The latent class analysis was based on the a priori hypothesis that the authors thought would be associated with the willingness to accept a new treatment. Five variables were tested for class membership: (1) a summary of experience of different treatment, (2) experience of the summary of different side effects, (3) the perception of the moral status of the embryo, (4) experience of advanced treatment, and (5) the importance of religion. A sum of how many treatments each respondent had was calculated, and also how many side effects they had experienced. Advanced treatment was based on treatment experience with one or more of apomorphine subcutaneous injection, apomorphine subcutaneous infusion, deep brain stimulation, levodopa-carbidopa intestinal infusion, and levodopa-entacapone-carbidopa intestinal infusion. The variable ‘the perception of the moral status of the embryo’ did not influence class membership and was therefore not included in the final class assignment model.

### Differences in respondents’ preferences

The statistical analyses of the preference data were based on a latent class model. A preference weight (i.e., coefficient) and a corresponding SE were estimated for all but one level of each attribute (i.e., reference attribute level) [21]. Dummy coding of the variables was selected for this analysis (i.e., corresponding to zero as the reference value). Each p-value is a measure of the statistical significance of the difference between the estimated preference weights for each level of the attribute compared to the reference attribute level. All results were considered statistically significant at *p* < 0.05. Confidence intervals (95%) were also provided for each preference weight. The Akaike information criterion (AIC) and the log-likelihood values were considered when selecting the appropriate model.

The latent class model was used to identify hidden (latent) classes of respondents’ preferences [22]. In latent class analysis, unobserved preference heterogeneity among respondents’ preferences is modeled as classes with similar preference patterns but with different variances across classes. Once preference patterns have been stratified into classes, the model determines the extent to which demographic characteristics impact the likelihood of belonging to a certain class. The systematic utility component (V) describes the latent construct that participant “r” belonging to class “c” reported for alternative A, B or C in choice task “t.” The final utility functions were as follows:

V_r,t,A&B|c_ = β1 * consist_hESC_r,t,A&B|c_ + β2 * consist_iPS_r,t,A&B|c_ + β3 * consist_electric_r,t,A&B|c_ + β4 * aim_slow_r,t,A&B|c_ + β5 * aim_repaire_r,t,A&B|c_ + β6 * know_500_r,t,A&B|c_ + β7 * know_5000_r,t,A&B|c_ + β8 * effect_50_r,t,A&B|c_ + β9 * effect_80_r,t,A&B|c_ + β10 * sideeffects_0.001_r,t,A&B|c_ + β11 * sideeffects_0.01_r,t,A&B|c_ + ε.

V_r,t,C|c_ = β1 * consist_drug_r,t,C|c_ + β2 ** aim_relief_r,t,C|c_ + β3 * know_5000_r,t,C|c_ + β4 * effect_50_r,t,C|c_ + β5 * sideeffects_0.01_r,t,C|c_ + ε.

A class assignment model was fitted after the specified utility function. The variables: experience in treatment, side effects, advanced treatment therapy and religious beliefs were tested for their potential impact on class membership in the model. The final class assignment function was:

V_n|c_ = β0 + β1* treatment_sum_|c_ + β2 * experience_sideeffects_|c_ + β3 * advanced_treatment_|c_ + β4 * Religion_dum_|c_ + ε.

### Relative importance of attributes

The relative importance of the attributes included in the DCE was calculated by estimating the difference in preference weights of the latent class model between the most preferred level of an attribute and the least preferred level of the same attribute [21]. The highest difference value was normalized to 1, which represents the most important value. The difference values were divided by the highest value to reveal the relative distance between all other attributes.

### Predicted acceptance uptake for treatment with hESCs

We calculated the predicted acceptance uptake for a potential treatment scenario using hESCs to treat patients with PD. Predicted acceptability can be understood as the probability that a participant will accept a described scenario. The scenario represents a hypothetical treatment scenario of treatments with hESCs based on the attributes assessment in the DCE. Attribute estimates assessed by the latent class model were used to calculate the predicted acceptability of attribute levels (treatment with hESCs, risk of severe side effects is 1 out of 1000 and 50 patients received treatment) in relevant future scenarios; (A) effect on symptoms is 2 out of 10, (B) effect on symptoms is 5 out of 10, and (C) effect on symptoms is 8 out of 10.

The predicted acceptability is presented as the percentage of 100 who would accept the presented scenario. The utility for the specific scenario was calculated by using the following equation:

V_Scenario 1_ = β_A_ + β_B_ + β_C_.

The predicted acceptability, the probability of accepting a specific scenario, was then calculated by using the following equation:

Predicted acceptance uptake = 1/(1 + exp^− V^_Scenario 1_).

## Results

### Respondent characteristics

The survey was completed by 498 respondents. Because the aim of this study to assess what factors influence preferences of patients with PD regarding stem cell-based therapies to treat PD in the future, the 43 respondents always selecting the ‘standard care’ alternative were excluded from the analysis. In total, 455 respondents were included in the final analysis. The mean age of the respondents was 66.7 years (SD 8.95). Most of the respondents were male (65.9%) and born in Sweden (90.3%) (Table 2), which mirrors the actual composition of this patient group [23]. Respondents’ demographic characteristics are presented based on their view on the moral status of an embryo. The majority (n = 252) viewed the embryo as merely a lump of cells; the others (n = 203) had the view that the embryo as something more than a lump of cells.

**Table 2:** Descriptive statistics of the respondents’ demographic characteristics presented as percentages, mean, or median with statistical testing between the different perceptions of whether the embryo is more than a lump of cells or only a lump of cells

	More than a lump of cells	Lump of cells	Total	
	(N = 203)	(N = 252)	(N = 455)	
				**P value** (**ANOVA**^**a**^)
**Age (years)**				
Mean (SD)	67.7 (8.86)	65.9 (8.96)	66.7 (8.95)	0.31
Median [Min, Max]	70.0 [40.0, 84.0]	67.5 [30.0, 84.0]	69.0 [30.0, 84.0]	
				**P value** **(Chi-square test)**
**Gender**				0.40
Female	74 (36.5%)	77 (30.6%)	151 (33.2%)	
Male	127 (62.6%)	173 (68.7%)	300 (65.9%)	
Other	2 (1.0%)	2 (0.8%)	4 (0.9%)	
**Country of birth**				0.45
Sweden	181 (89.2%)	230 (91.3%)	411 (90.3%)	
Other	22 (10.8%)	22 (8.7%)	44 (9.7%)	
**Occupational situation**				0.15
Employed	44 (21.7%)	77 (30.6%)	121 (26.6%)	
Retired	143 (70.4%)	172 (68.3%)	315 (69.2%)	
On sick leave	22 (10.8%)	17 (6.7%)	39 (8.6%)	
Jobseeker	3 (1.5%)	5 (2.0%)	8 (1.8%)	
Student	0 (0%)	2 (0.8%)	2 (0.4%)	
Other	3 (1.5%)	0 (0%)	3 (0.7%)	
				**P value** **(Kruskal-Wallis test)**
**Education**				0.11
No formal schooling	0 (0%)	1 (0.4%)	1 (0.2%)	
Elementary school	34 (16.7%)	29 (11.5%)	63 (13.8%)	
High school	50 (24.6%)	56 (22.2%)	106 (23.3%)	
Vocational training	22 (10.8%)	30 (11.9%)	52 (11.4%)	
University	97 (47.8%)	136 (54.0%)	233 (51.2%)	
**Health numeracy**				< 0.001
Low	57 (28.1%)	56 (22.2%)	113 (24.8%)	
Medium	82 (40.4%)	66 (26.2%)	148 (32.5%)	
High	64 (31.5%)	130 (51.6%)	194 (42.6%)	
**Health literacy**				0.040
Low	25 (12.3%)	24 (9.5%)	49 (10.8%)	
Medium	85 (41.9%)	88 (34.9%)	173 (38.0%)	
High	93 (45.8%)	140 (55.6%)	233 (51.2%)	
**Drug frequency**				0.58
Daily	201 (99.0%)	248 (98.4%)	449 (98.7%)	
1–6 time/week	0 (0%)	0 (0%)	0 (0%)	
1–3 times/month	0 (0%)	1 (0.4%)	1 (0.2%)	
Less than once/month	2 (1.0%)	2 (0.8%)	4 (0.9%)	
No drug	0 (0%)	1 (0.4%)	1 (0.2%)	
**Disease duration from established PD diagnosis**				0.48
0–6 month	5 (2.5%)	6 (2.4%)	11 (2.4%)	
6–12 month	10 (4.9%)	13 (5.2%)	23 (5.1%)	
1–3 years	32 (15.8%)	43 (17.1%)	75 (16.5%)	
3–5 years	34 (16.7%)	40 (15.9%)	74 (16.3%)	
5–10 years	52 (25.6%)	72 (28.6%)	124 (27.3%)	
10–15 years	35 (17.2%)	44 (17.5%)	79 (17.4%)	
15–20 years	16 (7.9%)	19 (7.5%)	35 (7.7%)	
More than 20 years	19 (9.4%)	15 (6.0%)	34 (7.5%)	
				**P value** **(ANOVA** ^**a**^ **)**
**Number of experienced side effects**				0.55
Mean (SD)	3.34 (2.94)	3.18 (2.77)	3.25 (2.85)	
Median [Min, Max]	3.00 [0, 16.0]	2.00 [0, 13.0]	3.00 [0, 16.0]	
				**P value** **(Chi-square test)**
**Any experience of advanced treatment**				0.69
No	158 (77.8%)	200 (79.4%)	358 (78.7%)	
Yes	45 (22.2%)	52 (20.6%)	97 (21.3%)	
				**P value** **(Kruskal-Wallis test)**
**Leftover embryos can be used to treat patients with PD**				< 0.001
Strongly agree	106 (52.2%)	209 (82.9%)	315 (69.2%)	
Agree	63 (31.0%)	34 (13.5%)	97 (21.3%)	
Undecided	26 (12.8%)	4 (1.6%)	30 (6.6%)	
Disagree	1 (0.5%)	0 (0%)	1 (0.2%)	
Strongly disagree	7 (3.4%)	5 (2.0%)	12 (2.6%)	
**Leftover embryos can be used to treat other diseases**				< 0.001
Strongly agree	86 (42.4%)	188 (74.6%)	274 (60.2%)	
Agree	72 (35.5%)	43 (17.1%)	115 (25.3%)	
Undecided	36 (17.7%)	13 (5.2%)	49 (10.8%)	
Disagree	2 (1.0%)	4 (1.6%)	6 (1.3%)	
Strongly disagree	7 (3.4%)	4 (1.6%)	11 (2.4%)	
**Leftover embryos can be used to treat diseases even if treatment with iPS cells are available**				< 0.001
Strongly agree	36 (17.7%)	130 (51.6%)	166 (36.5%)	
Agree	47 (23.2%)	50 (19.8%)	97 (21.3%)	
Undecided	64 (31.5%)	37 (14.7%)	101 (22.2%)	
Disagree	34 (16.7%)	21 (8.3%)	55 (12.1%)	
Strongly disagree	22 (10.8%)	14 (5.6%)	36 (7.9%)	
**Religion**				< 0.001
Not important	58 (28.6%)	143 (56.7%)	201 (44.2%)	
Slightly important	49 (24.1%)	43 (17.1%)	92 (20.2%)	
Moderately important	57 (28.1%)	49 (19.4%)	106 (23.3%)	
Important	25 (12.3%)	14 (5.6%)	39 (8.6%)	
Very important	14 (6.9%)	3 (1.2%)	17 (3.7%)	

^a^ANOVA: analysis of variance

### Attitudes of patients with Parkinson’s disease

**Fig. 2:**
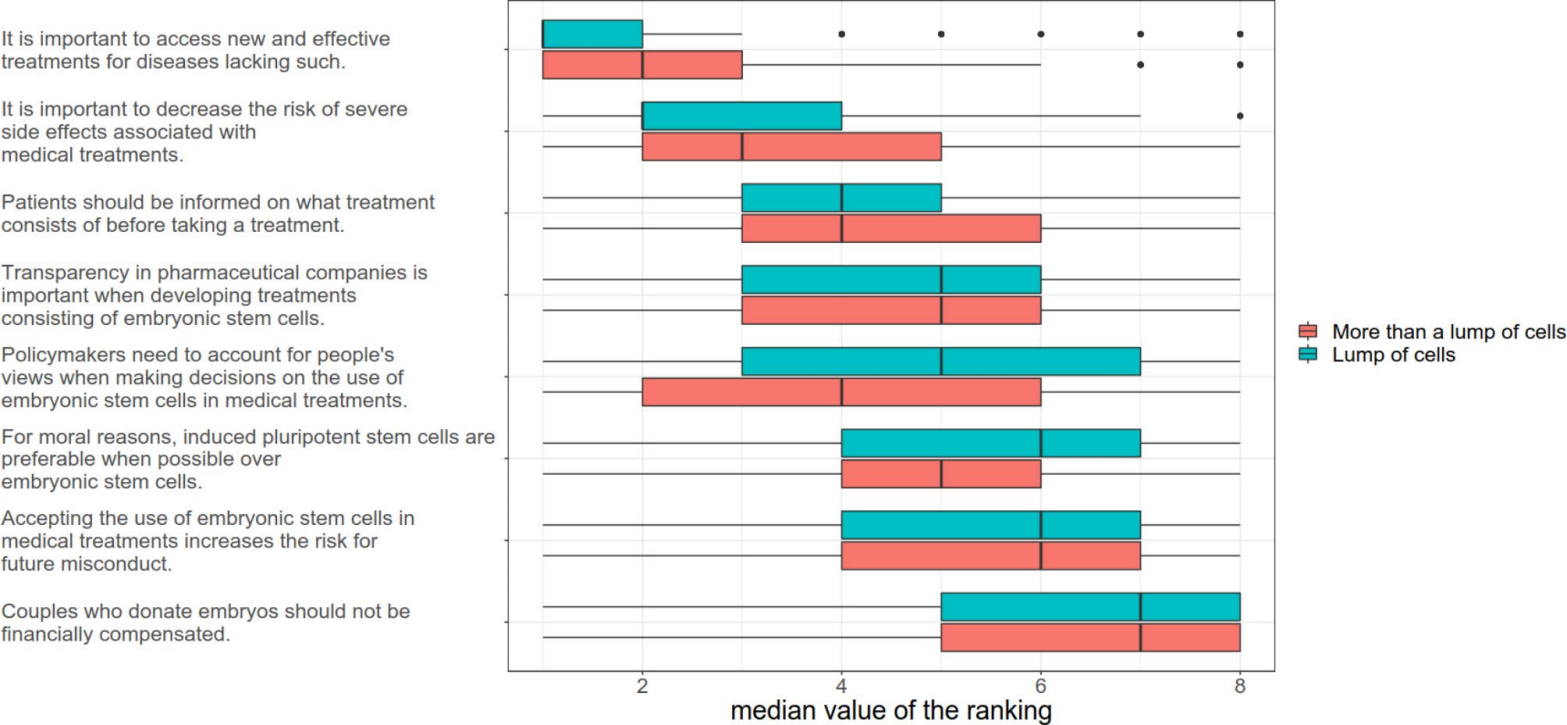
Attitudes of patients with PD. Bars indicate the values of the ranking of the statements on the left. Color bars in red and green dichotomize the participants’ view of a non-implanted embryo as a lump of cells. The dots indicate outliers, and the horizontal lines reveal the variability outside the upper and lower quartiles

Patients with PD ranked eight statements as part of a ranking exercise (Fig. 2). The results are presented in the subgroups “more than a lump of cells” and “lump of cells,” to reveal that the respondents’ rankings were not dependent on their views on the embryo. The highest ranked statement was “it is important to access new and effective treatment for diseases lacking such”; the second was “it is important to decrease the risk of severe side effects associated with medical treatments.”

### Treatment preferences of patients with Parkinson’s disease

The latent class model assessed three underlying preference patterns (classes) from data collected by the discrete choice survey (Table 3). The sign of the beta estimate reveals whether respondents were positive (> 0) or negative (< 0) about that attribute as compared to the reference level. Analysis of the data revealed that respondents have substantially different preferences regarding treatment with hESCs.

Respondents had an average probability of 38% of belonging to class 1. In class 1, drug (ref) was preferred over other treatment alternatives. Respondents preferred a treatment with the highest number of patients who have received treatment (n = 5000), greatest effectiveness (8 out of 10 will get enough effectiveness), and lowest risk of severe side effects (1 out of 1000). The likelihood of belonging to class 1 decreased (over the other levels of these attributes) for respondents with a higher number of experienced pharmacological treatments, experienced side effects and experience in advanced treatment. Religious beliefs increased the likelihood of belonging to class 1.

In class 2, with an average class probability of 23%, treatment with hESCs was preferred over iPS cells and electric stimulation. For aim of treatment, repairing damage caused by disease was preferred over slowing down disease progression. The highest number of patients who have received treatment, greatest effectiveness, and lowest risk of severe side effects (1 out of 1000) was preferred in class 2. Respondents with more experience with treatments are more likely to belong to class 2 as compared to class 1.

Treatment with hESCs was as the most preferred treatment alternative in class 3, over iPS cells and electric stimulation. Slowing down disease progression was as important as repairing damage caused by disease. The likelihood of belonging to class 3 increased for respondents with more experience in treatment, side effects, and advanced treatment. Religion (no) decreased the likelihood of belonging to class 3.

**Table 3:** Latent class analysis adjusted to class probability

Attribute	Levels	Class 1 Estimate	SE	CI (2.50–97.50)	Class 2 Estimate	SE	CI (2.50–97.50)	Class 3 Estimate	SE	CI (2.50–97.50)
Type of treatment										
	Drug (ref)									
	hESCs	-0.52**	0.17	-0.86–-0.18	1.28***	0.19	0.92–1.65	1.13***	0.11	0.91–1.36
	iPS cells	-0.64***	0.18	-0.98–-0.29	0.91***	0.18	0.57–1.26	1.09***	0.11	0.88–1.31
	Electric stimulation	-1.41***	0.26	-1.93–-0.89	0.61**	0.22	0.18–1.04	0.95***	0.17	0.60–1.31
Aim of treatment										
	Symptom relief (ref)									
	Slow down disease progression	0.31	0.20	-0.07–0.70	1.16***	0.25	0.67–1.65	1.08***	0.14	0.62–1.29
	Repair damage caused by disease	0.35	0.24	-0.13-0.82	1.66***	0.27	1.13–2.19	1.08***	0.15	0.81–1.34
Number of patients who have received the treatment										
	50 (ref)									
	500	0.39*	0.17	0.07–0.72	0.22**	0.12	-0.01–0.45	0.16	0.08	0.79–1.38
	5000	0.77***	0.17	0.44–1.09	0.37***	0.14	0.09–0.65	-0.16	0.09	-0.00–0.33
Effect on symptoms										
	2 in 10 (ref)									
	5 in 10	0.81***	0.22	0.37–1.24	1.87***	0.24	1.40–2.34	0.14	0.13	-0.33–0.02
	8 in 10	1.55***	0.27	1.01–2.09	3.14***	0.32	2.52–3.76	0.15	0.19	-0.12–0.40
Risk of severe side effects										
	1 in 1000	0.90***	0.20	0.50–1.29	2.13***	0.27	1.60–2.66	-0.04	0.14	-0.21–0.52
	10 in 1000	0.53**	0.18	0.18–0.89	0.92***	0.19	0.59–1.28	-0.15	0.10	-0.31–0.22
	20 in 1000 (ref)									
**Class probability model**										
Treatment experience (summary of pharmacological treatments tested)					0.26***	0.04	-0.18–0.34	0.13***	0.04	0.05–0.20
Experience with side effects (summary of all experienced side effects)					0.09***	0.02	0.04–0.14	0.13***	0.02	0.09–0.18
Experience with advanced treatment					1.21***	0.26	0.71–1.72	1.62***	0.24	1.16–2.09
Religion (yes or no)					-0.92***	0.17	-1.26–-0.59	-0.68***	0.14	-0.94–-0.40
Average class probability		0.38			0.23			0.37		
*,**,*** indicate significance at 10%, 5%, and 1%, respectively.										

### Relative importance of respondents’ preferences

**Fig. 3:**
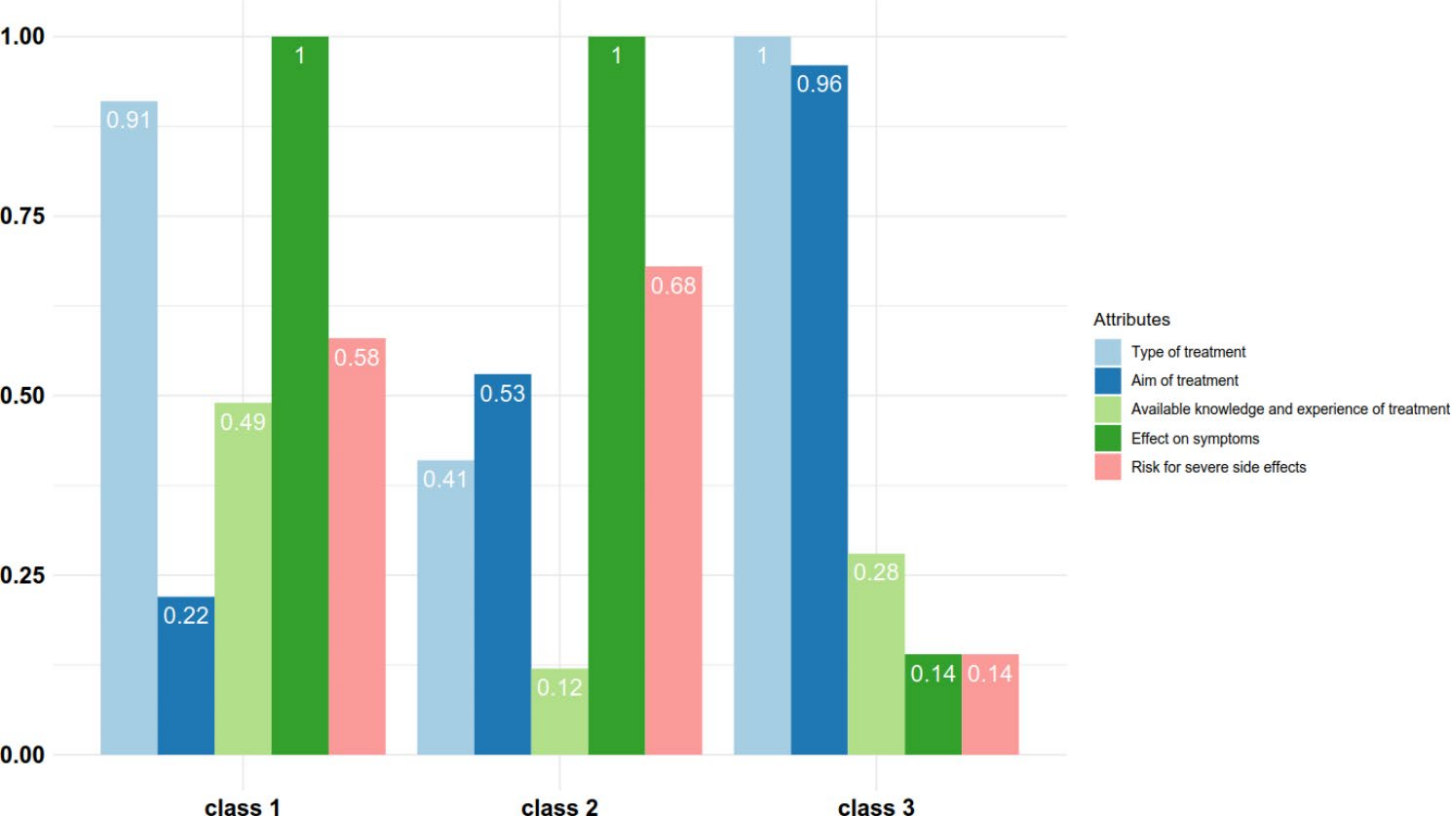
Relative importance scores of attributes

The most important attribute for class 1 was “effect on symptoms” (1), closely followed by “type of treatment” (0.91) (Fig. 3). The preference for hESC and iPS cells is lower compared to the reference ‘drug’. There is a statistically significant difference between the reference and hESC, iPScells and electric stimulation. Effect on symptoms was also the most important attribute in class 2, followed by the risk of getting a severe side effect (0.68) and aim of treatment (0.53). The most important attribute for class 3 was type of treatment. However, respondents preferred hESCs slightly more than iPS cells and electric stimulation compared to the reference drug. The aim of treatment was almost as important (0.96) as type of treatment.

### Predicted acceptance uptake

The predicted acceptance uptake for potential treatment scenarios in Parkinson’s treatment was based on treatment with hESCs for the different classes of the latent class model. The predicted uptake percentage was calculated for the group who would consider potential new treatments for PD (Table 4).

In this scenario, we assumed that treatment with hESCs was available for patients with PD in Sweden, the risk of severe side effects was 1 out of 1000, and 50 patients had previously received such treatment. In class 1, treatment with hESCs would be accepted by 94–95% (depending on the level of symptom relieve) if the effect on symptoms was 80%. The acceptance uptake would slightly increase in class 1 if the effect on symptoms was 50% (85–91%) or 20% (79–82%). The acceptance uptake for class 2 ranged from 85 to 100%. In class 3, 72–79% would accept the treatment scenario with hESCs if the aim was symptom relief, and 88–90% would accept the treatment scenario if the aim was to slow down disease progression or repair damage caused by disease.

**Table 4:** The predicted acceptance (latent class model) when risk of severe side effects is fixed at 1 out of 1000 and 50 patients received treatment for the different classes with the hESCs as the type of treatment

		hESCs (%)		
		Class 1	Class 2	Class 3
Effect on symptoms 2 out of 10				
	Symptom relief	79	98	72
	Slow down disease progression	81	99	88
	Repair damage caused by disease	82	100	88
Effect on symptoms 5 out of 10				
	Symptom relief	85	85	79
	Slow down disease progression	91	100	90
	Repair damage caused by disease	91	100	90
Effect on symptoms 8 out of 10				
	Symptom relief	94	100	75
	Slow down disease progression	95	100	90
	Repair damage caused by disease	95	100	90

## Discussion

Human embryonic stem cell-based therapies may soon become a reality for PD [7]. The ethical and policy issues need to be discussed along with scientific challenges to ensure that stem cell research and therapies are carried out in an ethically appropriate manner [24]. This DCE provides a fuller description of the relative importance of ethical concerns, values, and preferences among stakeholders, as well as conflicts between ethical views. For example, the DCE gives an understanding of the trade-off between effectiveness and use of hESCs. The results provide a perspective on ethical issues or risks and how they may be handled and/or minimized.

The aim of this study was to assess what factors influence preferences of patients with PD regarding stem cell-based therapies to treat PD in the future. This article reveals a substantial difference in respondents’ preferences observed in three latent preference patterns (classes). The first class revealed that “treatment effectiveness” closely followed by “type of treatment” was the most important attribute and that medications were preferred to other treatment alternatives. The second class also revealed “treatment effectiveness” to be the most important attribute. In this class, treatment with hESCs was preferred over other treatment alternatives. The third class mainly focused on type of treatment in their decision-making. They mostly preferred hESCs to iPS cells, electric stimulation, and medication.

These findings also correspond to findings from a recent qualitative study in Swedish patients with PD revealing that they were positive towards the use of hESCs for treatment of PD [25]. The study also reported that respondents found the treatment interesting and exciting regardless of whether iPS-cells were also available for treatment.

The class assignment model of the latent class analysis showed that respondents’ choices might be influenced by their experience in treatment, side effects, and advanced treatment therapy, and religious beliefs. Notably, preferences did not differ depending on view on the moral status of the embryo, as has been a major concern in ethical and legal debates regarding the use of leftover embryos [26]. Also, the importance of attitudes regarding what to do with the embryo was not associated with class membership.

Our study indicates that a great proportion (55%) of respondents perceived the embryo as just a lump of cells. The view of the moral status of an embryo did not differ based on respondents’ age, gender, country of birth, occupational situation, education, health numeracy, health literacy, drug frequency, disease duration, number of experienced side effects, or experience of advanced treatment. However, depending on respondents’ literacy and religion, their view on the moral status of the embryo differed significantly. A majority of those viewing an embryo as a lump of cells also reported high health numeracy and less importance of religious beliefs. Regarding the attitudinal questions, it was revealed that those who reported their moral view of the status of the embryo as a lump of cells also indicated that they strongly agreed to the use of leftover embryos for treatment of PD and other diseases, and to treat diseases even if treatment with iPS cells were available. Respondents viewing the embryo as something more than a lump of cells were more diversified in their attitudes. Moreover, the attitude ranking did not differ regarding respondents’ views on the embryo.

In summary, there is a difference in respondents’ views regarding the moral status of the embryo. Remarkably, patients with PD were not influenced by that difference. The moral status of embryos was not prioritized when making decisions regarding treatment with embryonic stem cells. As revealed in this study, respondents’ previous experience in treatment, side effects, advanced treatment, and religious beliefs influenced their preferences most.

This study has limitations, such as the relatively low response rate that may cause selection bias. Respondents who consistently chose the fixed alternative representing “standard treatment” in the DCE were excluded, as it was suspected that they had not understood the instructions to weigh different treatment characteristics against each other or that those respondents disliked the alternatives. Moreover, the attributes and levels were developed to represent potential future treatment alternatives for patients with PD. The group of respondents that always selected the ‘standard care’ was excluded from the main analysis to better understand the preferences of patients who would consider potential new treatments for PD. However, it was not possible to identify the reason behind the choice behavior. When comparing the characteristics of the “standard care” respondents that were excluded to others, there were no significant statistical differences. A suggestion for future research may be to include an open question to respondents mainly selecting the “fixed task” or “standard care” to better understand their choices. Another potential limitation of this study was that the DCE did not include a dominance test to test for the rationality in the choice behavior of the participants [27]. It is also worth noting that, qualitative interviews with participants could strengthen the conclusions of this study. Therefore, an implication for future research would be to plan for qualitative interviews already in the early planning phase of the research project.

Another possible limitation of this study may be that no interactions between attributes were posed to the DCE design, only conditions. Assuming the estimated response rate (N = 500), the study was not powered for interactions.

There is recent evidence on the preferences of professionals in the phase of prodromal treatment, shedding light on the difficulties in communicating risk-based information related to different hypothetical treatment option [28]. Previous preference elicitation of PD patients has only assessed preferences for attributes related to treatment with deep brain stimulation, pump assisted medication, or traditional oral medication [29]. To our knowledge, this is the first study to assess patients’ preferences for embryonic cell-based therapies to treat PD in the future. An advantage is also that it takes into regard the broader context of an ethical discussion related to the perceived moral status of human embryos. The significance of a study like this that also demonstrates predicted uptake of a new treatment is recently illustrated by the recent decision of the Swedish Medical Product Agency in 30th of November 2022 to give green light for the first clinical trial with hESC based therapy for patients with PD [27].

## Conclusions

The majority of the respondents would accept treatment with hESCs. Despite distinct differences in the perception of the moral status of an embryo, respondents’ preferences were not associated with it. Patient preferences may provide guidance in clinical decision-making and can inform ethical and legal guidelines for treatment with hESCs.

### Electronic supplementary material

Below is the link to the electronic supplementary material.

Supplementary Material 1

## Data Availability

The datasets generated and/or analyzed during the current study are not publicly available since it contains sensitive information, but are available from the corresponding author on reasonable request.
