# Supplementary material for: Patients accept therapy using embryonic stem cells for Parkinson’s disease: a discrete choice experiment
Source: BMC Med Ethics. 2023 Oct 12;24:83. doi: 10.1186/s12910-023-00966-1 (PMC10571417; doi:10.1186/s12910-023-00966-1)
Supplement: Supplementary file 1 — Supplementary Material 1 [file 12910_2023_966_MOESM1_ESM.pdf]

Supplementary file: Patients accept therapy using embryonic stem cells for Parkinson's disease: A discrete choice experiment

This is a translated version of the original survey that was in Swedish

Thank you for choosing to participate!

The survey takes about 30 minutes to answer and is divided into five different parts:

1. Background issues
   You must first answer a few questions about you and your background.

2. Information on how embryos can be used to treat diseases
   You can read through a short informational text to understand how embryos can be used in the treatment of Parkinson's disease.

3. Hypothetical choice situations
   In 8 different choice situations, you get to choose between three treatments against Parkinson's disease.
   The choice situations are hypothetical but realistic. We are interested to know which one
   treatment you prefer.

4. Rank and decide on some statements
   We ask a few questions about your views on the use of leftover, donated embryos.

5. Closing questions
   You get to answer a few questions about how it was to answer this survey

Part 1. Demographic questions

What do you identify as?

- Woman
- Man
- Other gender identity

How old are you? (year)

What is your country of birth?

What is your current occupation?

- Pensioner
- Sick leave
- Parental leave
- Job seeker
- Student
- None of the above options

What is your highest completed level of education?

- I have not completed any education
- Primary school/community school/real school
- Upper secondary school
- Vocational college or qualified vocational training
- University/college

How often do you use medication? Choose the answer option that best suits you.

- Daily
- 1-6 times per week
- 1-3 times per month
- Less often than once a month
- I never use drugs

How long have you been diagnosed with Parkinson's disease?

- 0-6 months
- 6-12 months
- 1-3 years
- 3-5 years
- 5-10 years
- 10-15 years
- 15-20 years
- More than 20 years

Have you received any of these treatments for Parkinson's disease? Click in all of them ever treated with, even if you are not using it today.

- Amantadine
- Apomorphine, injection pen (Apo-go Pen, Dacepton with D-mine pen)
- Apomorphine, medicine pump (Apo-go Pumpfill, Apomorphine,
- daceptone)
- Bromocriptine (Pravidel)
- Deep Brain Stimulation (DBS), surgery
- (electrical stimulation of the brain)
- Entacapone (Comtess, Entacapone)
- Cabergoline (Cabaser, Cabergoline, Dostinex)
- Treatment experience_8 Levodopa + benserazide (Madopark, Madopark Quick,
- Madopark Quick mite, Levodopa/Benserazide)
- Treatment experience_9Levodopa + entacapone + carbidopa, gel given via
- a pump with probe for the small intestine (Lecigon)
- Levodopa + carbidopa (Sinemet, Levocar, Flexilev)
- Treatment experience_11 Levodopa + carbidopa + entacapone (Dazonay,
- Levodopa/Carbidopa/Entacapone, Sastravi, Stalevo)
- Treatment experience_12Levodopa + carbidopa, gel given via a pump with
- tube to the small intestine (Duodopa, Lecigon)
- Pramipexole (Sifrol, Derinik, Mirapexin, Oprymea, Pramipexole)
- Rasagiline (Azilect, Rasagiline)
- Ropinirole (Adartrel, Requip, Ropinirole)
- Rotigotine, patch (Neupro)
- Safinamide (Xadago)
- Selegelin (Eldepryl)
- Tolcapon (Tasmar)
- None of the above options

Have you experienced any of the following side effects from your Parkinson's treatment(s).

disease?

- Involuntary movements (hypermobility)
- Depression
- Loss of appetite
- Hallucinations
- Excessive use of the drug
- Blood pressure changes
- Nausea
- Itching
- Liver effects
- Side effects_10Changed behavior: excessive gambling, excessive desire to buy,
- increased/changed sexual interest, binge eating

- Rash
- Anxiety
- Confusion
- Dizziness
- Diarrhea
- Local problems after pump use
- Local problems after DBS treatment
- Insomnia
- Sleep attacks
- Other side effect
- I have not experienced any side effects

Below are five statements about your current ability to understand and use information. Answer how well the statement is consistent with your ability.

- I can retrieve information from several different sources of information such as newspapers, the Internet, books, health care, family and friends.
- I can select the information I need from a variety of information sources.
- I can understand the information and share it with others.
- I can assess whether the information is credible
- With the help of the information, I can plan and decide what I need to do to improve my health

Part 2. Information on how embryos can be used to treat diseases

Below follows a short informational text. The text gives you a background for the election situations you will be faced in the next step.

Information on how embryos can be used to treat diseases

Stem cells

When a cell dies, it needs to be replaced. Then stem cells are needed. There are stem cells that are specialized to varying degrees. For example, there are specialized stem cells that create new ones blood cells. Other types of specialized stem cells are found in the brain and skin.

Embryonic stem cells (ES cells)

There are also stem cells that are not specialized, so-called embryonic stem cells (ES cells). They can divide any number of times and can, if they are cultivated and controlled in their development, replace basically any cell in the body. Using ES cells works researchers with developing cell-based treatments to treat diseases, for example Parkinson's disease.

The use of embryonic stem cells to develop medical treatment

To produce ES cells to develop treatments, cells from embryos are needed. One embryo is an egg that has been fertilized by a sperm. The embryos used have become over and donated by couples who have undergone in vitro fertilization (sometimes called IVF or in vitro fertilization), to get pregnant. When the cells are taken from the embryo, the embryo is destroyed.

The development of the ES cells can then be controlled, and the researchers can grow that kind of cells which is needed to develop a medical treatment. An embryo can give rise to one cell line which can then be used to develop treatment for various types of diseases, and for the treatment of many patients.

Continuation. Information on how embryos can be used to treat diseases

People with Parkinson's disease may benefit from the treatment Parkinson's disease is due to a lack of a signalling substance called dopamine. The lack of dopamine leads to other involuntary tremors, muscle stiffness, difficulty starting movements and that the movements take place more slowly. Other symptoms may be decreased sexual desire, constipation, urinary incontinence and depression. There is currently no cure for Parkinson's disease but there are treatments that can reduce the symptoms. By using ES cells to make cells that produce dopamine, researchers hope to avoid future deterioration and repair of damage that has occurred in people with Parkinson's disease.

Cells other than embryonic stem cells can also be used. Other cells can also be used to grow cells that produce dopamine. By taking specialized cells, to for example skin cells, and by modifying them, researchers can make them go backwards in development and become unspecialized again. Cells that have been modified in this way are called induced Pluripotent Stem Cells, or iPS cells. Just like embryonic stem cells, iPS cells can divide on itself any number of times and is believed to be able to develop into basically any kind of cell preferably. Researchers hope that even dopamine-producing cells developed from iPS cells will could be used to treat people with Parkinson's disease.

Pharmaceutical companies may make money from treatments with cells

Pharmaceutical companies work to produce and develop new treatments. A safe and effective treatment requires a long and costly development time. To pay for the development of pharmaceutical companies need to be able to make money from their products, even those that is based on donated skin cells and human embryos.

Part 3. Hypothetical choice situations

In this step you will be faced with 9 hypothetical choice situations. In every election situation, we want know which treatment you prefer.

We want you to imagine that you have no treatment against Parkinson's disease when choosing treatment. But you need treatment. Imagine you that you are otherwise in your current situation. Your doctor offers you to start treatment with one of the treatments presented. Which treatment do you prefer?

In each choice situation, you will be able to choose between three treatments against Parkinson's disease ("Operation A", "Operation B" and "Tablet or Pump Treatment"). Operation A and Operation B involves being admitted to hospital to undergo an operation on the brain. You are sedated during the operation. Tablet or pump treatment is given in the form of a tablet or using a pump. In pump treatment, drugs are pumped into the subcutaneous fat or the gut. Try to choose the option that you would prefer if you really had to choose between the three.

Instructions for understanding the hypothetical choice situations

In the choice situations, five characteristics of Operation A, Operation B and Tablet- or pump treatment is described. The characteristics will vary between the choice situations. For that you should understand what these characteristics mean, you will get one on the following pages brief description of the properties. If you are unsure while answering, you can place hover over the information you want to read more about to clarify what we mean. The different the properties are:

- What the treatment consists of
- The purpose of the treatment
- Available knowledge and experience about the treatment
- Effect on symptoms
- Risk of serious side effects

Description of the properties of the treatments

The treatment consists of...

Embryonic stem cells

Cells taken from donated fertilized eggs. The cells have been multiplied and directed to produce dopamine.

Induced pluripotent cells (iPS cells)

Your own or donated cells (eg blood cells) that have been propagated and directed to produce dopamine

Electrical stimulation

An implanted electrode with thin wire and stimulator that stimulates the brain

Drug

Drugs for Parkinson's disease

Description of the properties of the treatments

The purpose of the treatment is to...

Relef symptoms

Improve function and well-being without affecting the development of the disease. In step with the disease develops, doses usually need to be increased to obtain sufficient relief.

Slow the progression of the disease

Affects the development of the disease so that the disease develops more slowly than it had done if you have not been treated.

Repair damage caused by the disease

Affects disease progression and restores functions lost due to your Parkinson's disease.

Description of the properties of the treatments

The number who have previously received the treatment is…

The knowledge and experience that is available from a treatment depends on how many have been in the past received it. The total number who previously received the treatment is...

50 people

After clinical research studies, the treatment has been approved for treatment against Parkinson's disease. A total of 50 people have previously received the treatment.

500 people

After clinical research studies, the treatment has been approved for treatment against Parkinson's disease. In total, 500 people have previously received the treatment.

5000 people

After clinical research studies, the treatment has been approved for treatment against Parkinson's disease. In total, 5,000 people have previously received the treatment.

Description of the properties of the treatments

The percentage that gets a sufficient effect from the treatment is...

The treatment's effect on symptoms (e.g. balance difficulties, tremors, depression and dementia). The proportion that achieves sufficient function and well-being to at the moment not needing additional/different treatment for Parkinson's is...

2 out of 10 get sufficient effect

Out of 10 who receive the treatment, 2 people achieve sufficient function and well-being to occasion not needing additional/different treatment against Parkinson's

5 out of 10 get sufficient effect

Out of 10 who receive the treatment, 5 people achieve sufficient function and well-being to occasion not needing additional/different treatment against Parkinson's

8 out of 10 get sufficient effect

Out of 10 who receive the treatment, 8 people achieve sufficient function and well-being to occasion not needing additional/different treatment against Parkinson's

Description of the properties of the treatments

The risk of serious side effects is…

The risk that the treatment causes a serious side effect that produces a negative lasting effect the impact on function and well-being is…

1 in 1000

Out of a thousand people who start the treatment, 1 person suffers from some kind of serious side effect.

10 out of 1000

Out of a thousand people who start the treatment, 10 people suffer from some form of serious illness side effect.

20 out of 1000

Out of a thousand people who start the treatment, 20 people suffer some form of serious illness side effect.

Now you can start choosing! The first choice situation is presented on the next page.

Imagine that in your current situation you have no treatment for Parkinson's disease. But you need treatment. Your doctor offers you the treatments Operation A, Operation B and Tablet or pump treatment. Which treatment do you prefer?

You can have information explained to you by hovering over the words.

Task 1 out of 9 that was presented to each respondent

	**Operation A**	**Operation B**	**Tablet or pump treatment C**
The treatment consists of...	hESCs	iPS cells	Drug
The aim of the treatment is to...	Repair damage caused by disease	Symptom relief	Symptom relief
Number of patients that have received the treatment are...	5000 patients	50 patients	5000 patients
Treatment effect on symptoms (for example, balance difficulties, tremors, depression and dementia)...	2 out of 10	5 out of 10	5 out of 10
The risk that the treatment causes a serious side effect that has a negative lasting effect on function and well-being is...	20 out of 1000	10 out of 1000	10 out of 1000
	I select this alternative	I select this alternative	I select this alternative

Below are statements that you can agree with or disagree with. Tick that answer which best corresponds to your setting. Assume that the embryos in question have been left over fertility treatment and is donated by the couple for that very purpose, without financial compensation.

It is acceptable that embryos left over after fertility treatment…

a) ...used for treatment of Parkinson's disease.

b) ...used for the treatment of other types of diseases.

c) ...used for treatment of diseases, although induced pluripotent cells can be used with similar results.

Humans are perceived to have a special moral position in the sense of having rights just by being human. What a moral position one has human embryo that is only a few days old?

- The embryo is just a lump of cells, it's pointless to discuss it moral standing
- The embryo has a moral status that is in between just being one lump of cells and being a human.
- The embryo's moral status is closer to being a human being than just one lump of cells.
- The embryo has the same moral status as a human being.

How important is religion in your life?

- Very little importance
- Pretty little importance
- Neither big nor small importance
- Quite significant
- Great importance

Final questions

Did you fill in the questionnaire independently?

- Yes, I have filled in the survey on my own
- No, I have had the help of one or more people

How did you experience the information in this survey?

- Too much information
- Adequate amount of information
- Too little information

How did you feel about responding to the election situations?

- I was able to answer without difficulty

- I had to think for a while before I could answer
- I had a very hard time choosing

If you have any comments regarding the survey, you are welcome to write them here:

Thank you for your participation! We have now received your answers. If you have any questions regarding the study or want to get in contact us researchers, you are welcome to get in touch.
